# Chasing Immune Checkpoint Inhibitors in Ovarian Cancer: Novel Combinations and Biomarker Discovery

**DOI:** 10.3390/cancers15123220

**Published:** 2023-06-16

**Authors:** Ilaria Colombo, Katherine Karakasis, Sneha Suku, Amit M. Oza

**Affiliations:** 1 Oncology Institute of Southern Switzerland (IOSI), Ente Ospedaliero Cantonale (EOC), Via A. Gallino, 6500 Bellinzona, Switzerland; ilaria.colombo@eoc.ch; 2 Princess Margaret Cancer Centre, University Health Network, 610 University Avenue, Toronto, ON M5G 2M9, Canada; katherine.karakasis@uhn.ca (K.K.); suku.sneha@uhn.ca (S.S.)

**Keywords:** ovarian cancer, immune checkpoint inhibitors, immune therapy, tumor microenvironment, predictive biomarkers, anti-PD-1/PD-L1

## Abstract

**Simple Summary:**

Immune checkpoint inhibitors (ICIs) have been investigated in epithelial ovarian cancer in first-line and recurrent settings. When used as a single agent or in combination with chemotherapy, they have largely failed to improve patients’ outcome and thus, have not entered routine use in clinical practice. However, there are signs of promising activity in some early and late-phase clinical trials, combining immune checkpoint inhibitors with effective targeted agents, such as those targeting the tumor blood supply (antiangiogenics) or when taking advantage of impaired intra-cellular machinery that are unable to repair major cell damage (for example with poly-ADP-ribose inhibitors (PARP)). Further research is still needed to define predictive biomarkers that can identify patients more likely to respond to ICI combinations. New targets and treatment strategies are under investigation to define if and how immunotherapy can be incorporated into the treatment of epithelial ovarian cancer.

**Abstract:**

A deep understanding of the tumor microenvironment and the recognition of tumor-infiltrating lymphocytes as a prognostic factor have resulted in major milestones in immunotherapy that have led to therapeutic advances in treating many cancers. Yet, the translation of this knowledge to clinical success for ovarian cancer remains a challenge. The efficacy of immune checkpoint inhibitors as single agents or combined with chemotherapy has been unsatisfactory, leading to the exploration of alternative combination strategies with targeted agents (e.g., poly-ADP-ribose inhibitors (PARP)and angiogenesis inhibitors) and novel immunotherapy approaches. Among the different histological subtypes, clear cell ovarian cancer has shown a higher sensitivity to immunotherapy. A deeper understanding of the mechanism of immune resistance within the context of ovarian cancer and the identification of predictive biomarkers remain central discovery benchmarks to be realized. This will be critical to successfully define the precision use of immune checkpoint inhibitors for the treatment of ovarian cancer.

## 1. Introduction

Ovarian cancer represents the leading cause of death due to gynecological malignancies in developed countries, and about 19,710 new cases are estimated in the United States in 2023 [1]. Despite recent advances in our understanding of the underlying tumor biology and molecular characteristics of disease, the prognosis for women with epithelial ovarian cancer (EOC) remains poor due to a high incidence of recurrence and treatment resistance. Optimal cytoreductive surgery followed by platinum-based chemotherapy remains the gold-standard treatment of newly diagnosed EOC [2]. Maintenance treatment with the antiangiogenic agent bevacizumab in high-risk disease and/or with poly-ADP-ribose inhibitors PARP inhibitors (PARPis) have significantly improved patient outcome and are now incorporated in the standard treatment strategy of a first-line setting [2,3,4,5]. Nevertheless, many patients will inevitably experience disease recurrence or progression, and the success of the subsequent lines of therapy is challenged by the progressive occurrence of treatment resistance. Treatment options are represented by either a platinum-based combination or non-platinum chemotherapy, according to the sensitivity to the prior line of treatment (platinum-eligibility) [2,6]. Bevacizumab may be added to chemotherapy first-line, platinum-sensitive or platinum-resistant settings. In the recurrent setting, PARPis are also used as maintenance or in limited settings as a treatment strategy when not administered in first-line and according to genomic characteristics [2,4]. Recently, mirvetuximab soravtansine (an anti-folate receptor-α antibody drug conjugate) has been approved by the FDA for the treatment of platinum-resistant ovarian cancer [7]. No other targeted agents are available for the treatment of EOC, and there is an urgent need to discover new agents to improve patients’ outcome in first-line and recurrent settings.

Immune therapy and particularly immune checkpoint inhibitors (ICIs) have revolutionized cancer treatment and are now incorporated in the management of many solid tumors, including endometrial, cervical, melanoma, lung, head and neck, kidney and urothelial cancers, triple negative breast cancer and microsatellite unstable tumors [8,9,10,11]. However, their use as a single agent or in combination in EOC has been quite disappointing, and no immune therapy strategy is approved for the treatment of this malignancy. In this review, we will examine the literature on the tumor microenvironment and the evidence supporting the continued rationale for investigating ICIs in EOC, along with the available data on ICI efficacy as a single agent or in combination with chemotherapy or other target agents, and discuss the available biomarkers and their limitations.

## 2. Tumor Microenvironment in EOC

The tumor microenvironment (TME) plays a pivotal role in sustaining cancer cell proliferation, invasion and metastatic spread and influences anti-cancer treatment sensitivity or resistance [12]. Moreover, the immune component of the TME favors cancer cell elimination and is necessary to retain cancer cells from growing [12]. The recognition of tumor cells activates a T-cell mediated response, and a multi-step process with co-stimulatory signals result in tumor cell destruction [13,14]. Cancer cells evade the immune system through a negative regulation of the antigen presentation process, making cancer cells ‘invisible’ to the immune system, together with the activation of immunosuppressive pathways, to sustain immune tolerance. The complex mechanisms regulating the tumor immune response depend not only on the TME, but also on the host immune system, tumor genomic features, related inflammatory response, angiogenic processes and cytokine production [15].

Ovarian cancer is a recognized immunogenic tumor, and the presence of T cells within the epithelial component of the tumor (tumor-infiltrating lymphocytes—TILs) has an established prognostic role. Zhang et al. demonstrated an improved progression-free survival (PFS) and overall survival (OS), following debulking surgery and adjuvant chemotherapy in patients whose tumors were characterized by the presence of TILs, defined as CD3+ cells [16]. In the subgroup of patients with complete response after first-line treatment, the absence of TILs was associated with a significantly higher risk of disease recurrence compared to patients with TILs in the tumor samples (5 years OS 11.9% vs. 73.9%) [16]. A subsequent metanalysis further confirmed the prognostic role of TILs in EOC [17]. Other studies reported a positive association between intraepithelial CD8^+^ T-cells and CD8^+^/regulatory T-cells (Treg) ratio and survival in EOC [18]. CD8+ TILs vary among histological subtypes of EOC with the highest prevalence in high-grade serous ovarian cancer (83%, HGSOC) followed by low-grade serous (73%), endometrioid (72%), clear cell (52%) and mucinous subtypes (51%) [19]. *BRCA1* mutation, but not *BRCA2*, was associated with a higher level of CD8+ TILs [19]. As expected, due to the known heterogeneity of EOC, particularly HGSOC, there are differences in CD8+ TIL infiltration among different tumor sites [19]. Recent studies have attempted to explore the relevance of tumor heterogeneity in HGSOC by examining the malignant–immune interface in different samples from the same patients. Significant differences in the TME composition occurs among different sites of disease within the same patients, underlying the limitation of using a single site biopsy when studying the TME [20]. Other immune-infiltrating cells, such as Tregs, dendritic cells, myeloid-derived suppressor cells (MDSC) and macrophages play an essential role in suppressing anti-cancer immunity and maintaining self-tolerance [21,22,23].

**Figure 1:**
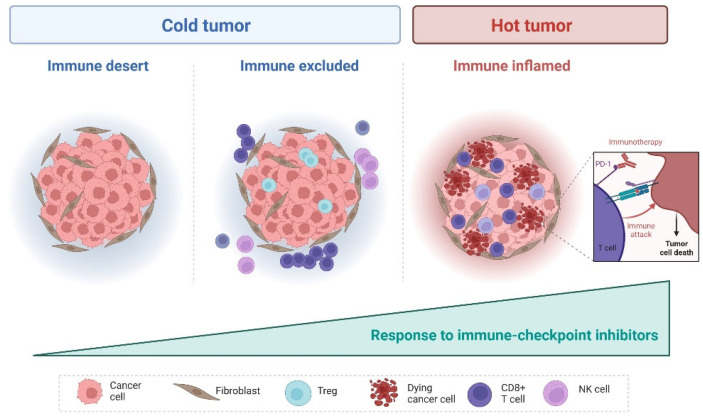
Immunophenotypes of ovarian cancer and their correlation with sensitivity to immune-checkpoint inhibitors. Created with Biorender.

Inhibition of the cytotoxic T-cell response is one of the main mechanisms used by cancer cells to evade the immune system and is mediated by several factors, such as the cytotoxic T-lymphocyte-associated protein 4 (CTLA-4), the programmed death receptor 1 (PD-1) and its ligand PD-L1, whose inhibition with ICIs constitutes one of the recent revolutions in cancer treatment [24,25]. The discovery of CTL-4 and PD-1 has fueled the development of many targeted agents and significantly improved patients’ outcome, becoming a part of the standard of care armamentarium across several cancer types [26,27,28,29]. Despite the remarkable progress, ICIs have shown limited effectiveness across all cancer types and even when an initial response is achieved, secondary resistance inevitably develops. The hallmarks of immune response have been described as key factors in governing the response and resistance to ICIs and include tumor intrinsic characteristics (tumor genome, epigenome and microenvironment), host immune system (systemic and antitumor immune response) and external factors (e.g., microbiota, age, infection) [30]. Three different immune phenotypes that correlate with sensitivity to ICIs have been proposed, following an analysis of tumor samples collected before treatment exposure [13]. The “immune-inflamed” phenotype is characterized by the infiltration of CD4+ and CD8+ T-cells within the tumor, PD-L1 expression in the tumor and high levels of pro-inflammatory cytokines [13]. It is assumed that a pre-existing immune response was initially present and then arrested as a consequence of the activation of inhibitory pathways and immune exhaustion. This results in immune tolerance and cancer growth, and thus, this subtype is more likely to respond to ICIs [31]. In the “immune-excluded” phenotype, immune cells are present but are confined in the stroma surrounding the tumor cells, preventing the activation of an adequate antitumoral immune response and, as a consequence, the efficacy of ICIs might be reduced [13]. The “immune-desert phenotype” is characterized by the lack of TILs within the tumor and the stroma, reflecting the absence of a pre-existing T-cell immune response (Figure 1). As a result, ICIs might not elicit an adequate tumor response in this setting [13]. Moreover, it has also been shown that an abundance of TILs alone is not sufficient to support an adequate immune response and ICI efficacy. A reduced cancer cell–lymphocytes interaction or the lack of an adequate immune recognition might also underscore ICI failure [20].

## 3. Immune Checkpoints in EOC

**Figure 2:**
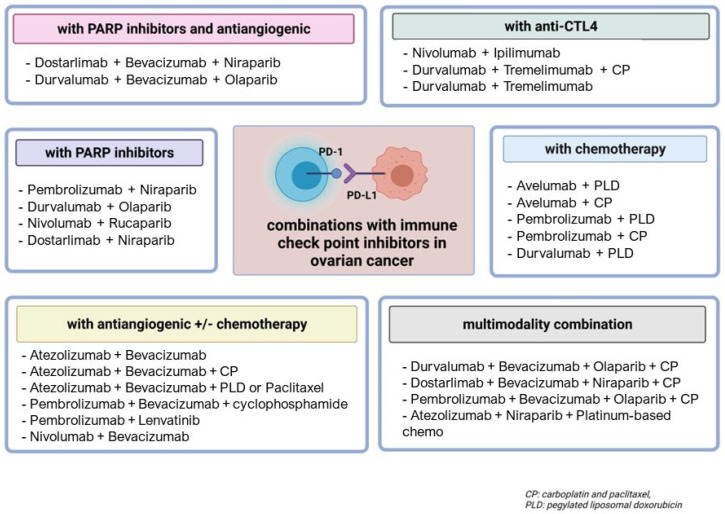
Combinations of immune checkpoint inhibitors under investigation for the treatment of ovarian cancer.

Anti-PD-1/PD-L1 agents have been investigated for the treatment of EOC in different settings, such as first-line, platinum-sensitive or platinum-resistant recurrence, and strategies explored thus far have included single-agent or combination treatment or maintenance approaches (Figure 2). The most investigated immune checkpoint inhibitors in EOC are the anti-PD-1 pembrolizumab, nivolumab and dostarlimab, the anti-PD-L1 durvalumab and the anti-CTL4 ipilimumab.

### 3.1. Single-Agent Strategy

Immune checkpoint inhibitors have been investigated as single agents in the recurrent setting with limited signs of activity and without biomarkers of response identified (Table 1). The activity of the anti-PD-L1 avelumab was assessed in patients with recurrent EOC, and an overall response rate (ORR) of 9.6% was reported across different histological subtypes [32]. Notably, two patients with clear cell ovarian carcinoma have been enrolled and both had a partial response (PR) [32]. No correlation with PD-L1 expression measured on either available archival tissue on tumor cells or on immune-infiltrating cells was observed [32]. Similar results were obtained with the anti-PD-1 nivolumab and pembrolizumab [33,34,35]. The KEYNOTE-100 study aimed to investigate the activity of pembrolizumab in a large cohort of patients with recurrent EOC and to explore the role of PD-L1 expression, measured as the combined positive score (CPS), as a predictive biomarker of response [36,37]. The overall response to pembrolizumab was modest in two cohorts of patients, selected according to the number of previous lines of treatment. No clinical features, such as platinum-free interval, the number of previous lines of treatment and histology, were predictive of response. This study identifies a positive correlation between a higher PD-L1 expression measured as a CPS score (the combined measure on tumor cells, lymphocytes and macrophages) ≥10 and the ORR, while previous reported studies have assessed PD-L1 only on tumor cells and did not show any correlation with efficacy [36,37].

Although PD-L1 in EOC is known to be associated with a poor prognosis and suppression of CD8+ T-cell [57], the lack of the activity of single-agent ICIs in EOC and the absence of correlation between ICI efficacy and PD-L1 expression suggests that the PD-1/PD-L1 pathway might not be the prevalent mechanism of immune evasion in EOC, or there are other suppressors of immune function which have yet to be identified.

### 3.2. Combination Strategies

Given the complexity of the mechanism of immune evasion adopted by cancer cells, a simultaneous inhibition of different pathways relevant for tumor cell proliferation and progression may represent a successful approach to increase treatment performance. Thus, as performed in many other cancer types, ICIs have been added to the different available standard or investigational agents to examine their activity in the setting of newly diagnosed or recurrent EOC (Table 1).

#### 3.2.1. ICIs with Chemotherapy

Chemotherapy remains the cornerstone of treatment for EOC in first-line and in the recurrent settings. Different preclinical and clinical findings have supported the rationale of combining chemotherapy with immunotherapy agents to enhance antitumor activity [58]. When cancer cells are exposed to some chemotherapy agents, negative effectors of the immune response such as Tregs and MDSC are depleted [59,60]. Chemotherapy also induces tumor cross-antigen presentation, stimulating an immunogenic cell death [61,62].

Preclinical data showed that doxorubicin promotes antigen presentation and increases T-cell infiltration, representing a promising partner for immunotherapy combinations [63]. In platinum-resistant EOC, pegylated liposomal doxorubicin (PLD) was combined with the anti-PD-1 agent pembrolizumab [39] or the anti-PD-L1 agents avelumab [41] or durvalumab [64], without significant signs of improved efficacy (Table 1). The combination of PLD and pembrolizumab achieved an ORR of 26% in a small group of patients, but no predictive biomarkers to identify patients who are more likely to benefit from this treatment were identified [39]. The expression of PD-L1 on tumor and inflammatory cells, the presence of TILs and the T-cell inflamed gene expression profile (GEP) did not correlate with patient outcome when assessed on archival tissue [39]. Moreover, the presence of *MYC* amplification was reported to induce an immunosuppressive microenvironment sustained by an increased expression of PD-L1 [65], and preliminary results reported a reduced activity of durvalumab–PLD combination in tumors harboring *MYC* amplification [64].

Two phase 3 studies combining avelumab with chemotherapy did not met the primary endpoint of improving median progression free survival in either the recurrent or first-line setting [41,42]. The phase 3 JAVELIN200 study failed to show an improvement in the addition of avelumab to PLD via PFS in recurrent platinum-resistant EOC [41]; however, a dual biomarker analysis suggested a possible benefit of the combination in the subgroup of patients with PD-L1 positive (≥1% tumor cell, ≥5% immune cells or both) and CD8 positive (≥1% immune cells) tumors, warranting further exploration in future trials [41]. Notably, the JAVELIN100 trial investigating the role of avelumab in combination and/or following platinum-based chemotherapy for previously untreated EOC failed to meet the primary endpoint of improved PFS at the preplanned interim analysis and was prematurely interrupted [42].

In the neoadjuvant setting, pembrolizumab has been investigated in combination with carboplatin and paclitaxel for four cycles, followed by interval debulking surgery and standard adjuvant treatment (+/− bevacizumab) with or without pembrolizumab up to 2 years in a phase 2 randomized trial, and the results have been presented at the 2021 American Society of Medical Oncology (ASCO) conference [43]. The addition of the immune checkpoint inhibitor resulted in a small increase in ORR and complete cytoreduction rate without difference in median PFS. Biomarker analyses on tumor tissue and liquid biopsy are ongoing and will be of utmost relevance, given the possibility to compare treatment naïve baseline samples with samples collected at the time of interval debulking surgery, following exposure to ICIs, and the possibility to explore possible correlations between BRCA and homologous recombination deficient (HRD) status, tumor mutational burden (TMB), PD-L1 expression and immune signatures [43].

Thus, as of today, the available data do not support the addition of ICIs to standard chemotherapy in EOC, and other treatment strategies have been explored to define if a role for ICIs in EOC might still be foreseen.

#### 3.2.2. ICIs with Antiangiogenic Agents

The presence of crosstalk between immune and endothelial cells within the TME has underscored the rationale to combine ICIs and antiangiogenic agents in different disease settings [28,66,67,68,69]. Rapidly growing tumors are characterized by hypoxia that upregulates the hypoxia-inducible factor 1 (HIF-1) and as a consequence, the vascular endothelial growth factor (VEGF), which induces abnormal vascularization, leading to immune evasion [70]. The presence of malfunctional blood vessels induces immunosuppression through different mechanisms such as an increased expression of PD-L1, a reduced infiltration of cytotoxic T-cells but an increased activation of Tregs and an infiltration of MDSC [71,72]. Nevertheless, immune cells play a critical role in regulating angiogenesis with Tregs promoting angiogenesis [73], whereas CD8+ T-cells suppress endothelial cells proliferation through the interferon-γ pathway [74].

Following the positive results reported from several phase 3 trials in first-line and recurrent settings, the anti-VEGF agent bevacizumab has been incorporated into the standard of care armamentarium for the treatment of EOC [75,76,77,78]. Based on the available preclinical data on its synergistic effect with immunotherapy, different combinations have been tested [44,45,46,47]. When combined with atezolizumab in a small population of patients with platinum-resistant ovarian cancer, it has shown a tolerable safety profile and limited activity (ORR 15%), but some durable responses were reported with no correlation with PD-L1 expression, as observed in other trials of ICIs in EOC [44]. The phase 3 IMagyn50 trial investigated the addition of atezolizumab to the standard of care bevacizumab and platinum chemotherapy as a first-line treatment of FIGO stage III and IV EOC [45]. Consistent with findings from the JAVELIN100 trial [42], study findings here did not show an improvement in PFS in the intention-to-treat population or in the PD-L1 positive group (≥1%) where PD-L1 positivity was based on expression on immune cells [45]. A non-preplanned exploratory analysis examined differences in PFS in patients with PD-L1 ≥ 5%, and a trend toward PFS improvement was observed [45]. Whilst the limited sample size of this subgroup does not support a defined conclusion, these findings support toward a further analysis of the efficacy of ICIs in patients with high PD-L1 EOC. Further analysis is ongoing to identify potential biomarkers of response and to explore if any group of patients, such as patients with *BRCA* mutation or HRD tumors, might have a more favorable outcome with the addition of ICIs to standard treatment.

More recently, the results of the phase 3 ATALANTE trial, which investigated the combination of atezolizumab, bevacizumab and platinum-based chemotherapy in a platinum-sensitive recurrent setting, have been presented. As observed with other similar combinations, this trial also did not meet its primary endpoint and no PFS benefit was observed, regardless of PD-L1 expression [49].

The antiangiogenic multikinase inhibitor lenvatinib has been combined with pembrolizumab in an ongoing phase 2 basket study (NCT03797326), including a cohort of patients with recurrent OC, where an intriguing ORR of 32.3% was observed when used as the fourth line of treatment [48]. The study is still ongoing and confirmatory results in an expanded cohort are necessary to confirm the efficacy of this combination as well as the identification of predictive biomarkers to guide patient selection.

#### 3.2.3. ICIs with PARP Inhibitors

PARPis represent one of the major paradigm shifts in the treatment of EOC in first-line and recurrent settings and are now part of the standard of care [3]. The main antitumor activity of PARPis is elicited through the inhibition of base excision repair arming the capability of cancer cells to repair single strand DNA break [79]. In cells with *BRCA* mutation or HRD, this results in cell death according to the well-described mechanism of synthetic lethality [79]. In addition, the accumulation of unrepaired DNA breaks leads to a cytosolic release of DNA fragments that activate a stimulator of interferon genes (STING) innate immune response pathway and ultimately the activation of a cytotoxic T-cell response [80,81]. Preclinical data demonstrate that PARPis upregulate PD-L1 expression [82]. Together with the increased expression of neoantigens when the DNA repair mechanism does not function properly, these data provided the rationale for combining ICIs and PARPis to enhance their antitumoral activity.

The combination of niraparib and pembrolizumab showed a limited efficacy in a small phase 1/2 trial (TOPACIO) in a heterogenous population of patients with recurrent platinum-resistant or platinum-ineligible EOC [50]. The reported ORR was 18% in the whole population, with no differences among *BRCA*-mutated or wild-type tumors or according to HRD status [50]. However, it is known from previous studies that the efficacy of PARPis in platinum-resistant *BRCA* wild-type tumor is limited [83,84] as the activity of single-agent anti-PD-1/PD-L1 [33,36]. Thus, the ORR reported in the platinum-resistant *BRCA* wild-type and in the non-HRD population (ORR 19% in both) might underscore the synergistic effect of these two agents in this difficult-to-treat population [50].

In patients with germline *BRCA 1/2* mutated platinum-sensitive recurrent EOC, olaparib combined to durvalumab provided an ORR of 71.9% with a median duration of response of 10.2 months, which was better than when this combination was offered as second-line treatment [51]. In patients with *BRCA 1/2* wild-type tumors, the ORR was 34.4% with a median duration of response of 6.9 months [52]. Despite the fact that these promising results provide a biological rationale for combining ICIs and PARPis, confirmatory large trials with a control arm are warranted to define if such a chemotherapy-free regimen can be considered as an option for patients with platinum-sensitive recurrent EOC. Moreover, a careful evaluation of the safety profile of these combinations and the possible long-term toxicities should be considered [85].

#### 3.2.4. Multimodality Combination

Multimodality combinations assessing PARPis, antiangiogenics and ICIs administered simultaneously are feasible given the non-overlapping toxicity profile of these agents. The phase 2 MEDIOLA trial investigated double and triple combinations in *BRCA*-mutated and *BRCA* wild-type PARPi naïve patients with platinum-sensitive recurrent EOC [51,52]. The efficacy and safety results of combining olaparib, bevacizumab and durvalumab in patients with *BRCA* wild type were initially presented at the 2020 European Society for Medical Oncology (ESMO) conference [52]. The triplet combinations showed promising activity with a confirmed ORR of 77.4% and a duration of response (DOR) of 11.1 months. An exploratory analysis focused on the genomic instability score (GIS), performed trough Foundation Medicine^®^ next-generation sequencing (NGS) on tumor tissue. The GIS was defined as positive if the loss of heterozygosity (LOH) score was ≥14 and a mutation in *BRCA 1/2* or in another DNA repair genes (e.g., *ATM*, *BRIP1*, *PALB2*, *RAD51C*, *BARD1*, *CDK12*) were present. No difference was observed among GIS positive and GIS negative patients; the combination of durvalumab and olaparib induced a similar ORR in *BRCA*-mutated patients but a lower ORR (50%) in GIS positive in the same setting [51]. More recently, OS data have been reported with a median OS of 31.9 months with the triplet combination and 26.1 months with the combination of olaparib and durvalumab [51]. Given the small sample size, a conclusion cannot be drawn these results do raise the question of whether the addition of the antiangiogenic agents could increase genomic instability and thus sensitize GIS negative tumors to these agents [52]. An improved understanding of the role of the addition of bevacizumab in the setting of *BRCA*-mutated subjects is also much required.

Another triple combination with dostarlimab, bevacizumab and niraparib showed interesting signs of activity with an ORR of 17.9% in a small cohort of patients with platinum-resistant OC [53]. No differences in efficacy were observed among *BRCA* or other homologous recombination repair genes mutations [53].

Ongoing clinical trials are now investigating the role of the triplet combination of PARPis, antiangiogenic and ICIs in first-line maintenance setting. This multimodality strategy is of particular interest for HRD negative tumors, that are usually less sensitive to PARPis. Recently, positive results in PFS at an interim analysis of the DUO-O trial (NCT03737643), investigating durvalumab plus olaparib added to platinum-based chemotherapy and bevacizumab, have been announced [86]. Additional details are eagerly awaited. Relevant ongoing clinical trials with ICIs in EOC are reported in Table 2.

#### 3.2.5. Combinations of ICIs

The combination of anti-PD-1/PD-L1 with anti-CTL-4 is another effective strategy that has been pursued with positive results in different solid tumors, showing an increased efficacy with a dual checkpoints inhibition [26,27,87,88]. The combination of nivolumab and ipilimumab has been investigated in a phase 2 randomized trial in patients with recurrent EOC with a platinum-free interval (PFI) < 12 months [54]. Compared to nivolumab alone, the combination yielded a better ORR (31.4 versus 12.2%) with a limited PFS of 3.9 vs. 2 months [54]. Patients with clear cell histology achieved a more durable clinical benefit, as reported in other trials [33]. Similar to other ICIs, no correlation with PD-L1 expression in the tumor or in the immune cells was observed and no other potential biomarkers of efficacy were explored.

The combination of nivolumab and the anti-CTL-4 tremelimumab, as sequential versus concomitant administration, in platinum-resistant heavily pretreated patients with HGSOC, did not show signs of activity, having reported no objective response and limited PFS [56].

A small phase 2 study presented at the 2021 Annual Meeting of the Society of Gynecology Oncology (SGO) investigated the combination of durvalumab and the anti-CTL-4 tremelimumab in combination with neoadjuvant chemotherapy. All 23 enrolled patients achieved an objective response with a complete resection to no residual disease at the interval debulking surgery in almost 74% of the patients [55]. The neoadjuvant setting provided a unique opportunity for biomarker exploration, given the availability of baseline tissue at the time of first diagnosis and tumor samples from the interval debulking surgery. Comparing the pre- and post-chemoimmunotherapy samples, the tumor microenvironment switched toward a more inflamed phenotype [55]; however, correlation with treatment efficacy is not yet available, and further analysis and a longer follow-up—other than efficacy confirmation in a larger population—are required to better define the potential role of the double ICIs combined with chemotherapy for the treatment of EOC.

## 4. Clear Cell Ovarian Cancer

Clear cell ovarian cancer (CCOC) is a rare histological subtype, with a variable incidence according the geographic areas and ethnicity [89]. If compared to the more frequent HGSOC histotype, it is characterized by worse prognosis and chemoresistance [90,91]. Clear cell ovarian cancer frequently harbors molecular aberration in the switch/sucrose nonfermentable ATP-dependent (SWI/SNF) chromatin remodeling complex and the AKT/PI3K/mTOR pathways, while alterations in TP53 or homologous recombination genes are infrequent, as copy number variations. [92,93,94].

Microsatellite instability (MSI) is a well-known predictive biomarker of response to ICIs regardless of tumor type and, as a result, pembrolizumab has received a histology-agnostic approval for MSI-high tumors [95]. While MSI is a very rare feature of HGSOC, it is seen more frequently in endometrioid or CCOC [96]. CCOC with MSI have an enhanced immunogenicity, a higher number of TILs and increased expression of PD-L1 [97], and thus, may represent a good candidate for ICI treatment. In fact, complete and durable responses to ICIs have been reported in patients with CCOC [32,33,36,45,98]. An exceptional response to pembrolizumab has been reported in a patient with heavily pretreated CCOC [99]. Interestingly, a whole exome sequencing analysis demonstrated a PD-L1 genomic rearrangement, causing an aberrant PD-L1 expression [99]. Moreover, the combination of anti-PD1 agent sintilimab and bevacizumab achieved a promising ORR of 40% in a small cohort of recurrent CCOC treated with at least one prior line of chemotherapy [100].

A randomized multicenter phase II trial is ongoing to investigate the efficacy and safety of durvalumab versus physician-choice chemotherapy in patients with recurrent clear cell ovarian cancer (NCT03405454) and may provide better evidence of the role of anti-PD-1/PD-L1 in this specific histological subtype [101].

## 5. Predictive Biomarkers of ICI Activity

Different biomarkers predictive of ICI efficacy or resistance have been explored in many tumor types with the aim of better selecting those patients that could most benefit from these agents and spare exposure to unnecessary toxicity in patients who would not have a predicted improvement in tumor response or survival. Many elements drive responses to ICIs, such as the tumor microenvironment milieu, the tumor genetic signature, the host immune system and environmental factors [15].

PD-L1 expression on either tumor cells or tumor-infiltrating immune cells has emerged as one of the first biomarkers undergoing investigation. Regulatory approval for the use of ICIs is limited to a specific threshold of PD-L1 expression in some tumor types (e.g., non-small cell lung cancer, cervical, bladder cancer or triple negative breast cancer) [102]. However, many limitations remain as a result of different companion diagnostics used for immunohistochemistry analysis: the type of cells analyzed (tumor cells or immune cells or a combined scored) and cut-offs implemented that vary among tumor types, clinical trials and ICIs used [102].

In EOC, randomized phase 3 trials to date have not demonstrated a benefit with the addition of an ICI to standard treatment [41,45,49,103]. Exploratory biomarker analysis identified a subset of patients with a specific PD-L1 expression cut-off with a trend toward improved ORR or PFS. In the IMagyn50 trial, a trend toward a better PFS was observed in the subgroup of patients with PD-L1 expression ≥5% on immune-cells, receiving atezolizumab in addition to standard of care first-line chemotherapy [45]. In the JAVELIN200 study, an improvement in PFS was reported when avelumab was added to PLD in the subgroup of patients with PD-L1 ≥ 1% on tumor cells or ≥5% on immune cells [41]. Similarly, in the KEYNOTE-100 trial single agent pembrolizumab showed signs of activity in patients with a higher PD-L1 score [37]. Despite these encouraging findings, caution must be exercised in interpreting the results, given the small sample size, exploratory nature of the analysis and different assays used. Findings do raise the question whether the identification of a correct threshold for PD-L1 positivity is necessary to define those patients that could benefit from an ICI. It is also necessary to consider the different distribution of PD-L1 expression in patients with EOC and a low prevalence of high PD-L1 positivity. In the JAVELIN-200 trial, an improvement in PFS was also observed in patients with CD8+ positive tumors defined as ≥1% CD8 expression on immune cells [41]. Data show a greater benefit in tumors that were both PD-L1 and CD8 positive, which suggests that patients with an ‘inflamed’ tumor phenotype might be the population in which further exploration of ICIs is warranted, despite being a minority of patients with recurrent EOC [41].

Genomic aberrations continue to emerge as potential predictive biomarkers for targeted treatment and immune therapy. Microsatellite instability is a well-established biomarker of response to ICIs and pembrolizumab has been granted approval in patients with germline or sporadic genomic aberrations in mismatched repair genes [95,104]. This genomic feature is not commonly found in EOC, and particularly in the more common high-grade serous or endometrioid histological subtypes. Nevertheless, microsatellite instability might occur in clear cell or low-grade tumors [105]. Another tumor genomic feature that has emerged as a predictive biomarker in response to ICIs is high tumor mutational burden [106]. This is based on a likely higher immunogenicity of tumors that express greater neoantigens, resulting from deficient DNA repair machinery [106]. Whilst tumor mutational burden correlates with tumor response in some tumor types, for example melanoma, lung or bladder cancers, it has failed to do so in other tumor types [107,108]. Although the predictive role of tumor mutational burden remains controversial, it is well established that HGSOC does not harbor a high mutational load, but copy number variations are more prevalent, and this might support the lack of a striking efficacy of ICIs observed in this tumor type [105]. Approximately 50% of HGSOC have an HRD associated with defect in the DNA double strand break repair pathway, the accumulation of DNA damage and high replication stress [109]. Despite this, HRD tumors do not show a higher tumor mutational burden and thus, this feature itself does not correlate with response to ICIs.

Whilst the role of the tumor microenvironment or genomic features in influencing the activity of ICIs remains to be well-identified, there is increasing research to investigate the role of gene expression signatures in tumor or in tumor-infiltrating immune cells. These may in future provide a better insight into those features that correlate with response to immunotherapy.

## 6. Discussion

Over the last decade, immunotherapy and particularly ICIs, have improved outcome in many cancers. Practice changing clinical trials have shown an improvement in patients’ outcome when anti-PD-1/PD-L1 have been used as a single agent or in combination with chemo or targeted therapy in different malignancies and have become the standard of care in many settings [27,28,29,67]. Following these promising results and the acknowledgment of the role of the immune microenvironment in cancer progression, ICIs have been explored in numerous clinical trials in EOC. Despite these efforts, results have not supported the incorporation of immunotherapy as standard treatment strategies in EOC [41,45]. Biological rationale supported the combination with chemotherapy, antiangiogenic agents and PARPis, and some signal-seeking trials have raised expectations that a multimodality approach could overcome the intrinsic resistance of EOC, but results from confirmatory trials are urgently needed [50,51,52].

It has also to be recognized that in single-agent and combination clinical trials, some patients responded to an ICI-based treatment with reports of single cases of long-lasting clinical benefit and ‘exceptional’ responders; however, it is evident that many questions remain [32,36,99]. Which tumor or clinical features might explain sensitivity to ICIs? Are there a minority of patients who would benefit from immunotherapy? Did some trials include an enriched population more likely to respond to ICIs, as CCOC, MSI-H or *BRCA*-mutated tumors? Why do most patients with EOC not respond to ICI treatment? Many similar questions remain unanswered and there is an urgent unmet need to understand the biological mechanisms that sustain this resistance. Deep translational analysis on tumor tissue and liquid biopsy collected before and during an ICI-based treatment are of utmost importance to investigate the tumor microenvironment and its changes, following exposure to treatment, as well as postulating potential predictive biomarkers of activity or defining the mechanism of intrinsic resistance [35,110]. Particularly, these analyses should be incorporated in the design of early phase clinical trials and will require collaborative efforts to collect clinical and translational data of exceptional responders, which will improve the understanding of rarely observed sensitivity to ICIs in patients with EOC. Moreover, a longitudinal collection of patient samples along the disease trajectory might help in better understanding the evolution and the heterogeneity of this disease and trials are ongoing (NCT03419689, NCT03420118). Novel high-throughput technologies such as single cell RNA sequencing, multiplex immunohistochemistry, multifunctional assays of immune cell components and genetic signatures might also provide further insight into the cancer biology and support the discovery of novel predictive biomarkers [111].

Another relevant aspect for discussion is in identifying which is the most reliable endpoint to be used in clinical trials assessing the ICI efficacy. In phase 2 trials, ORR is typically used as a primary endpoint, but it is still uncertain if this is the best surrogate for ICI efficacy, given that the pattern of response to ICIs differs from the one commonly observed with chemotherapy or target agents [112,113]. Moreover, a higher-than-expected efficacy of chemotherapy administered as a subsequent line after failure of an anti-PD1/PDL-1 regimen has been reported [114,115,116,117,118,119]. It has been hypothesized that immunotherapy might induce a modification in the tumor microenvironment, favoring a better response to subsequent lines of chemotherapy. Thus, new trial designs investigating sequential strategies instead of multiple combination requires further exploration.

Furthermore, we still need to identify which immunotherapy agents beyond ICIs warrant investigation in EOC, and the drug development field is actively exploring new opportunities. Tumor vaccines [120], adoptive T-cell therapies, chimeric antigen T-cell receptors (CAR-T), bispecific antibodies [121] or engineered cytokines [122] are some examples. The combination of a survivin vaccine with pembrolizumab and low-dose cyclophosphamide showed early signs of activity in a phase 1 trial in advanced recurrent EOC [120]. TILs are not the only immune cells present in the tumor microenvironment of EOC, and myeloid-derived suppressor cells and macrophages are also highly represented, for which trials are ongoing with agents targeting these immune cells.

Upon the identification of promising agents, a personalized approach selecting the best immune-oncology drug tailored to the patient’s immune milieu at a specific time-point could be an ideal and promising strategy to target the cancer-specific immune evasion. This will require a real-time conduct of clinical and translational research in parallel to fast-track knowledge from the bench to the bedside to improve the outcome of women diagnosed with advanced EOC.

## 7. Conclusions

Improving immunotherapeutic approaches for ovarian cancer is an urgent priority. Initial studies were relatively disappointing, and these have led to a careful evaluation of the impact of the tumor microenvironment in modulating immune responses and the careful assessment of combination strategies. These seem to be demonstrating benefits, and very early signals from some studies have suggested improvement in progression-free survival in the first-line setting.

The importance of patient selection, personalized treatment and the identification of the optimal target and the best combination regimen are of utmost importance in furthering the development of immunotherapy in EOC. Collaborative initiatives for translational and clinical trials are strongly needed to expedite progress in this field and ultimately improve the care of women with ovarian cancer.

## Figures and Tables

**Table 1:** Main clinical trials of ICIs as single agent or combination in EOC.

First Author/ Study Name	Agents	Phase	Setting	Histology	Biomarkers for Pts Inclusion	*n*	ORR	mPFS (Months)	Exploratory Biomarkers
**Single agent**									
Disis et al. [32] JAVELIN Solid Tumor	Avelumab	1b	PSOC/PROC median previous lines: 3	all	no	125	All: 9.6% PSOC: 3.6% PROC:5.3%	2.6 (95% CI, 1.4–2.8)	- PD-L1 in tumor cells (archival tissue) PD-L1 > 1%: ORR 11.8% PD-L1 neg: ORR 7.9% PD-L1 > 5%: 12.5% - PD-L1 in immune cells PD-L1 > 10%: ORR 0% PD-L1 neg: ORR 12.2% - BRCA: no correlation
Hamanishi et al. [33]	Nivolumab	2	PROC	all	no	20	15%	3.5 (95% CI, 1.7–3.9)	PD-L1 in tumor cells (archival tissue): no correlation
Omatsu et al. [38] NINJA	Nivolumab vs. gemcitabine or PLD	3	PROC	all	no	316	8 vs. 13%	2.0 vs. 3.8, HR 1.5, (95% CI: 1.2–1.9)	PD-L1: no correlation BRCA status: no correlation
Varga et al. [34] KEYNOTE-028	Pembrolizumab	1b	PSOC/PROC	all	PD-L1 ≥ 1% in tumor and immune cells	26	11.5%	1.9 (95% CI, 1.8–3.5)	NA
Colombo et al. [35] (INSPIRE-ovarian cohort)	Pembrolizumab	2	PSOC/PROC	HGSOC	no	21	0%	1.9	PD-L1: no correlation Other: under investigation
Matulonis et al. [37] KEYNOTE-100	Pembrolizumab	2	PSOC/PROC 2 cohorts: (A) 1–3 prior lines (TFI 3–12 months) (B) 4–6 prior lines (TFI > 3 months)	all	no	Cohort A 285 cohort B 91	A + B: 8.5% A: 8.1% B: 9.9%	2.1 in both cohorts (95% CI, cohort A 2.1–2.2 and cohort B 2.1–2.6))	PD-L1 as CPS score (archival tissue) in both cohorts CPS < 1: ORR 5% CPS ≥ 1: ORR 8% CPS ≥ 10: ORR 13.8%
**Combinations**									
**Chemotherapy**									
Lee et al. [39]	Pembrolizumab + PLD	2	PROC	all	no	26	26.1%	5.6 (95% CI 1.7–10.1)	PD-L1 archival tissue, MPS: no correlation T-cell inflamed GEP score: no correlation
O’Cearbhaill et al. [40]	Durvalumab + PLD	1/2	PROC	all	no	40	22.5%	5.5 (95% CI 0.3 to 28.8+)	MYC amplification (resistance)
Pujade-Lauraine et al. [41] JAVELIN200	Avelumab + PLD vs. PLD vs. avelumab	3	PROC	all	no	566	Ave + PLD: 13% PLD: 4% Ave: 4%	Ave + PLD 3.7 (95% CI 3·3–5·1) PLD 3.5 (2·1–4·0), HR 0.78 Ave 1.9 (1.8–1.9), HR 1.68	PD-L1 pos (≥1% tumor cells or ≥5% immune cells) and CD8 pos (≥1% immune cells), archival tissue: trend toward better PFS.
Monk et al. [42] JAVELIN100	Platinum-based chemotherapy + avelumab, followed by avelumab maintenance vs. platinum-based chemotherapy, followed by avelumab maintenance vs. platinum-based chemotherapy	3	First-line	all	no	998	Ave combination 36% vs. Ave maintenance 30% vs. 30%	Ave combination 18.1 (95% CI 14·8-NE), HR 1.14 Ave maintenance 16.8 (13·5-NE), HR 1.43 PLD NE	NA
Ray-Coquard et al. [43] NeoPembOv	Carboplatin + paclitaxel +/− pembrolizumab	2	Neoadjuvant	HGSOC	no	91	73.3% vs. 62.1% Rate or complete resection: 73.8% vs. 70%	19.3 (95%CI 15–24.5) vs. 20.8 (17–23.4)	NA
**Antiangiogenic (+/− chemotherapy)**									
Moroney et al. [44]	Atezolizumab + bevacizumab	1b	PROC	all	no	20	15%	4.9 (range 1.2–20.2)	PD-L1: no correlation
Moore et al. [45] IMagyn 50	Carboplatin + paclitaxel + bevacizumab + atezolizumab/placebo	3	First-line	all	PD-L1 on immune cells (1% vs. ≥1%), stratification factor	1301	93 vs. 89%	19.5 vs. 18.4, HR 0.92 (95% CI, 0.79–1.07)	PD-L1 ≥ 1%: PFS 20.8 vs. 18.5 months (95% CI, 0.65 to 0.99) PD-L1 > 5%: PFS NR vs. 20.2 months
Zsiros et al. [46]	Pembrolizumab + bevacizumab + cyclophosphamide	2	PSOC/PRSOC	all	no	40	47.5%	10 (95% CI 1.3–5.7)	NA
Liu et al. [47]	Nivolumab + bevacizumab	2	PSOC/PROC	all	no	38	28.9%	9.4 (95% CI, 6.3–14.7)	- PD-L1 on tumor cells: PD-L1 < 1%: ORR 45% PD-L1 ≥ 1%: ORR 14.3%
Lwin et al. [48] LEAP005 (ovarian cohort)	Lenvatinib + pembrolizumab	2	PSOC/PROC (4L)	all	no	31	32.3%	4.4 (95% CI 4.0–8.5)	NA
Kurtz et al. [49] ATALANTE	Carboplatin-based chemotherapy+ bevacizumab+ atezolizumab/placebo	3	PSOC	all (non-mucinous)	no	614	NA	13.5 vs. 11.3 HR 0.83 (95% CI 0.69–0.99)	PD-L1 ≥ 1%: PFS 15.2 vs. 13.1 months, HR: 0.86 (0.63–1.13)
**PARP Inhibitors**									
Konstantinopoulos et al. [50] TOPACIO	Niraparib + pembrolizumab	1/2	PROC or platinum ineligible	all	no	62	18%	3.4 (95% CI, 2.1–5.1)	- PD-L1 pos (CPS score): PD-L1 < 1%: ORR 45.5% PD-L1 ≥ 1%: ORR 14.3% - tBRCA and HRD: no differences
Drew et al. [51] MEDIOLA (doublet)	Olaparib + durvalumab	2	PSOC	*gBRCA* mutant	no	32	71.9%	11.1 (95% CI 8.2–15.9)	
				*gBRCA* wild type	no	32	34.4%	5.5 (95% CI 3.6–7.5)	Genomic instability status (GIS) GIS-pos: ORR 50% GIS-neg: ORR 16.7%
**PARP inhibitors + antiangiogenic**									
Drew et al. [52] MEDIOLA (triplet)	Olaparib + durvalumab + bevacizumab	2	PSOC	gBRCA wild type ≤2 prior lines of chemo	no	31	87.1%	14.7 (95% CI 10–18.1)	Genomic instability status (GIS) GIS-pos: ORR 100% GIS-neg: ORR 75%
Liu et al. [53] OPAL (cohort A)	Dostarlimab + bevacizumab + niraparib	2	PROC	High grade or carcinosarcoma ≤2 prior lines	no	41	17.9%	7.6 (95% CI 4.2–10.6)	PD-L1 as CPS score: CPS pos (≥1%): ORR 15% CPS neg (<1%): ORR 22% t*BRCA* status: *BRCA* mutant: ORR 25% *BRCA* wild type: ORR 16%
Double ICIs									
Zamarin et al. [54] NRG-GY-003	Ipilimumab + nivolumab vs. nivolumab	2	PFI < 12 months ≤3 prior lines	all	no	100	31.4 vs. 12.2 %	3.9 vs. 2, HR 0.53 (95% CI, 0.34 to 0.82)	- PD-L1 in tumor cells (archival tissue) PD-L1 pos: ORR 40 vs. 33.3% PD-L1 neg: ORR 26.9 vs. 20% - PD-L1 ≥ 1% in immune cells PD-L1 pos: ORR 30 vs. 31.2% PD-L1 neg: ORR 27.6 vs. 21.4%
Lee et al. [55] KGOG 3046/TRU-D	Carboplatin and paclitaxel + durvalumab + tremelimumab	2	Neoadjuvant	all	no	23	100% No residual disease after surgery: 74%	NA	NA
Hinchcliff et al. [56]	Durvalumab + tremelimumab concomitant vs. sequential	2	PROC	HGSOC	no	61	8.7 vs. 0%	1.87 (95% Ci 1.77–2.17) vs. 1.84 (95% CI 1.77–2.43)	NA

N: Number; pts: patients; PSOC: platinum-sensitive ovarian cancer; PROC: platinum-resistant ovarian cancer, ORR: overall response rate; mPFS: median progression-free survival; PD-L1: programmed death ligand 1; PLD: pegylated liposomal doxorubicin; MPS: modified percentage score; GEP: gene expression profile; ICIs: immune checkpoint inhibitors; NA: not available; TFI: treatment free interval; Ave: avelumab; NE: not estimated; NR: not reached; tBRCA: tumor BRCA; HRD: homologous recombination deficiency; gBRCA: germinal BRCA; HGSOC: high-grade serous ovarian cancer; CI: confidence interval.

**Table 2:** Main ongoing phase 3 clinical trials investigating ICI combinations in EOC.

Study	Agents	Setting	Histology	*n*	Biomarkers for Inclusion/Stratification	NCT Number
KEYLYNK-001/ ENGOT Ov43/ GOG3036	CP +/− bevacizumab + pembrolizumab/placebo + olaparib/placebo	First-line	*BRCA* wild type	1284	PD-L1 (CPS > 10): stratification	NCT03740165
FIRST/ ENGOT Ov44	CP +/− bevacizumab + niraparib + dostarlimab/placebo	First-line	mucinous and low-grade excluded	1228	PD-L1: stratification factor	NCT03602859
ATHENA/ ENGOT Ov45	Rucaparib/placebo + nivolumab/placebo	Maintenance after first-line	Mucinous excluded	1000	HRR status by mutation analysis	NCT03522246
DUO-O/ ENGOT Ov46	CP + bevacizumab + durvalumab/placebo + olaparib/placebo	First-line	High grade	1104	BRCA status *	NCT03737643
ANITA/ ENGOT Ov41/ GEICO 69-O	Platinum-based chemotherapy + atezolizumab/placebo + niraparib	Recurrent PSOC	High-grade serous or endometrioid *≤*2 prior lines	414	BRCA status: stratification	NCT03598270
NItCHE-MITO33	Niraparib + dostarlimab vs. chemotherapy of physician choice (+/− bevacizumab)	Recurrent non platinum eligible ≤2 prior lines	all	427	PD-L1 and HRD status: stratification	NCT04679064
AGO-OVAR 2.29/ ENGOT Ov34	PLD or paclitaxel + bevacizumab + atezolizumab	Recurrent PROC ≤3 prior lines	all	664	PD-L1: stratification	NCT03353831

CP: Carboplatin + paclitaxel; PSOC: platinum-sensitive ovarian cancer; PLD: pegylated liposomal doxorubicin; CPS: combined positive score; HRR: homologous recombination repair. * *BRCA*-mutated patients will be enrolled in a single arm part (*n* = 150) of the trial and will receive durvalumab and olaparib.

## Data Availability

Not applicable.
